# A Berlin-sided retrospective of the origins of metabolic control theory

**DOI:** 10.1098/rsfs.2023.0024

**Published:** 2024-02-09

**Authors:** Tom A. Rapoport

**Affiliations:** Harvard Medical School and Howard Hughes Medical Institute investigator, Boston, MA, USA

**Keywords:** Berlin, sided, retrospective, origins, metabolic control theory, metabolism

## Abstract

Metabolic control theory (MCA) is celebrating its 50th anniversary. The theory introduced quantitative terms that describe the importance of an enzyme for the regulation of the overall flux and of metabolite concentrations. MCA was developed independently by two groups. The Berlin group included Reinhart Heinrich, Tom A. Rapoport and Samuel M. Rapoport, and the Edinburgh group Henrik Kacser and James A. Burns. Here, I provide a brief reminiscence from the perspective of the Berlin group.

Metabolism was a hot field in the early 1970s (and is popular again today), but it was (and still is) also very confusing. This can be confirmed with a casual glance at a metabolic chart. How are these thousands of metabolites regulated? What role do individual enzymes play in regulating the flux through the various pathways? These were questions that stimulated research in many laboratories, including the one headed by my father Samuel Rapoport in East Berlin. Dad was particularly interested in glycolysis, as he was studying red blood cells that had essentially no other metabolic pathway. His coworkers, particularly Gisela Jacobasch, had measured the changes of glycolytic metabolites under many different conditions, so there was a rich dataset available. What was missing was an understanding of why these changes occurred. My father realized that a deeper understanding of metabolite and flux regulation required a theoretical approach.

It was a lucky coincidence that Reinhart Heinrich had just finished his PhD on solid state physics in Dresden. Reinhart had heard a presentation by Manfred Eigen at a Leopoldina meeting and had decided that biology was ripe for a physicist. He travelled to Berlin and took his chances to show up at the Biochemistry Institute. He bumped into my dad in front of the institute and was invited to his office. After a short discussion, Reinhart was offered a job on the spot, and he immediately accepted. He was given a very small room with no windows. This is where I met him towards the end of my PhD in 1971, and we hit it off right away. We started discussing how we could develop a theory of metabolic regulation. Reinhart had no prior experience with biology and thus relied on me, and vice versa: my knowledge in maths was far behind his. However, I knew enough to understand his equations and appreciate their beauty, as I had attended a special high school in science and maths and had done some mathematical modelling during my PhD. I remember that I had a little notebook, into which I scribbled the first ideas on how to express the changes of a flux through a pathway in response to changes of the activity of an individual enzyme in the pathway. Reinhart then suggested that this should be expressed as the derivatives of the natural logarithms.

Over the next months, Reinhart and I developed the theory that is now called ‘metabolic control analysis’ (MCA) (we originally called it ‘control theory’). The major concept was simple and it was gratifying that analytical expressions could be derived for linear enzymatic pathways. We demonstrated that previous terms, such as ‘rate-limiting enzyme’, were inappropriate in most situations, as rate control was usually shared among several enzymes. We also showed that the ‘cross-over theorem’, in which enzymes are identified for which metabolite changes before and after have different signs, is inadequate to identify effectors of a metabolic pathway or rate-controlling enzymes. Finally, we applied the theory and mathematical modelling to glycolysis in red blood cells, which led to the conclusion that the overall flux is controlled by hexokinase and phosphofructokinase.

We first presented our work at a meeting in Oberhof in 1972, in the south of East Germany. Neither Reinhart nor I had given a talk in English before. I remember that we were coached by my dad on how to pronounce the words ‘glycogenolysis’ and ‘gluconeogenesis’ in the introductory sentence. The talks by Reinhart and me were a great success. The meeting was attended by known scientists in the field, including David Garfinkel, who congratulated us on the achievements.

We initially summarized our work in a paper in an East German journal [1], but then submitted three full papers to the *European Journal of Biochemistry*. This turned out to be an ordeal. Since we needed several copies of the manuscripts for submission, we decided to type them on a matrix that could then be used in a ‘spirit duplicator’. In this copying system (the only one available in East Germany), the matrix sheet was fastened onto the drum of the machine and served as a template for the copies. Because alcohol was used to transfer the ink, the resulting copies smelled as if straight from a distillery. What was worse, the ink faded away quickly. And the reviewing process indeed took several months! The papers were initially rejected, but luckily for us, the editor-in-chief, Claude Liebecq, noticed that the negative reviewer Joe Higgins had not accepted a single paper during his tenure on the editorial board. So, he decided to overrule Higgins' report. When the paper went into print, the copy editors were in despair. Not only had the ink faded away, but they were also not used to the many equations and super- and subscripts. It took several snail mailings back and forth to make the corrections.

Then came the shock when we discovered that Henry Kascer and Jim Burns had developed essentially the same theory [2]. It must have been a shock for them too. I have seen this happen over and over again: it seems that when a discovery is ripe, it will be made by several people at the same time. I am happy to say that there was mutual respect between the Berlin and Edinburgh groups, although it took a while to agree on a common nomenclature. Our main publication is still my most cited paper [3]. Indeed, many systems biologists consider the development of MCA one of the earliest contributions to the field.

Over the following years, I repeatedly collaborated with Reinhart, often on projects originating from my experimental work. Unfortunately, after 36 years of collaboration and deep friendship, he died suddenly in 2006 at the age of 60. The last paper we published provided an explanation for how organelles maintain different protein compositions despite communicating by bidirectional vesicle transport [4]. Although not much cited, I still believe that this paper provides an answer to a fundamental problem.

I am the only person of the original MCA developers still alive. I left the field a long time ago, but I am happy to see that the theory is still alive and valued. This special edition is testimony to the fact that MCA has evolved and is being applied to different metabolic pathways. So, happy birthday MCA, and many wishes for a successful future!

## Data Availability

This article has no additional data.
